# Demographics, in-hospital analysis, and prevalence of 33 rare diseases with effective treatment in Shanghai

**DOI:** 10.1186/s13023-021-01830-4

**Published:** 2021-06-08

**Authors:** Xiaoshu Cai, Georgi Z. Genchev, Ping He, Hui Lu, Guangjun Yu

**Affiliations:** 1 Center for Biomedical Informatics, Shanghai Children’s Hospital, Shanghai, China; 2 SJTU-Yale Joint Center for Biostatistics and Data Science, Shanghai Jiao Tong University, Shanghai, China; 3 Department of Bioinformatics and Biostatistics, Shanghai Jiao Tong University, Shanghai, China; 4 Bulgarian Institute for Genomics and Precision Medicine, Sofia, Bulgaria; 5 Shanghai Hospital Development Center, Shanghai, China

**Keywords:** Rare disease, Orphan disease, Health care policy, Shanghai, China, Epidemiology, Rare disease prevalence

## Abstract

**Background:**

Rare diseases are ailments which impose a heavy burden on individual patients and global society as a whole. The rare disease management landscape is not a smooth one—a rare disease is quite often hard to diagnose, treat, and investigate. In China, the country’s rapid economic rise and development has brought an increased focus on rare diseases. At present, there is a growing focus placed on the importance and public health priority of rare diseases and on improving awareness, definitions, and treatments.

**Methods:**

In this work we utilized clinical data from the Shanghai HIE System to characterize the status of 33 rare diseases with effective treatment in Shanghai for the time period of 2013–2016.

**Results and conclusion:**

First, we describe the total number of patients, year-to-year change in new patients with diagnosis in one of the target diseases and the distribution of gender and age for the top six (by patient number) diseases of the set of 33 rare diseases. Second, we describe the hospitalization burden in terms of in-hospital ratio, length of stay, and medical expenses during hospitalization. Finally, rare disease period prevalence is calculated for the rare diseases set.

## Background

### Rare diseases: a global public health burden and challenges

Rare diseases are ailments which impose a heavy burden on individual patients and society as a whole [1]. Quite often, the total number of patients suffering from a specific rare disease is only a few tens or hundreds of people, however, rare diseases, considered holistically, are not so rare after all—in the United States, around 30 million Americans are afflicted with one of the ~ 7000 such diseases. There is no universally accepted definition what constitutes a rare disease [2]. The United States Orphan Drug Act of 1983 defines rare diseases as those that affect fewer than 200,000 individuals; the European Union defines them as diseases that affect less than 5 in 10,000 individuals, whereas the World Health Organization (WHO) defines rare diseases as those with prevalence in the range of 0.65–1‰.

Most rare diseases are of genetic etiology, and effective treatments exist for only a few of them [3]. The rare disease management landscape is not a smooth one—a rare disease is quite often hard to diagnose, treat, and investigate. For individual suffering from a rare disease the journey from symptoms, to diagnosis, and to treatment is arduous and difficult one. Firstly, receiving an accurate diagnosis may be a long-drawn process driven by the lack testing modalities and existing knowledge and information due to the rarity of the condition. Having overcome the hurdle of accurate diagnosis, the patient may face the obstacle of lack of treatment, or if the treatment is not included in a government established health insurance scheme—a financially ruinous self-funded cost of treatment [4]. From governance and health care perspective, while certain regions are well-advanced in many aspects of rare diseases management, in many others there is a lack of comprehensive policy, or a national rare disease plan and legislative framework that is specifically aimed to address the complex web of issues surrounding rare diseases is still in development or yet to be fully implemented [5].

### Focus on China

China, as the most populous country in the world, offers its own unique perspectives. China’s rapid economic rise and development has also brought an increased focus on rare diseases. At present, there is a growing importance placed on the management of rare diseases and their inclusion as a public health priority. A current push is in place for improving awareness, definitions, and treatments, and there is a rise in current interest to search for a comprehensive healthcare policy solutions and research. In a recent development, in the last decade medical treatments for certain rare diseases such as Pompeii disease, Gaucher’s disease and others have been included as covered treatment in Shanghai and other locations, however rare diseases continue to impose a significant economic burden on Chinese society and individuals [6, 7].

While expert consensus indicates that a rare disease could be defined as one with incidence of < 1 in 500 000 [8], a formal legislative definition of rare disease is not yet fully developed [9] and alternative approaches such as rare disease lists in lieu of prevalence-based definitions have recently emerged [10]. The first local list of rare diseases was released by the Shanghai Health and Family Planning Commission, the list titled “List of Major Rare Diseases in Shanghai” [11] consists of 56 rare diseases with effective treatment (Shanghai List). In 2016, the National Rare Disease Registry of China was implemented. Furthermore, representing a major milestone, in 2018, China’s First National Rare Disease List (National List) was promulgated by the following five national governmental bodies: the Ministry of Science and Technology, the State Drug Administration, the Ministry of Industry and Information Technology, the National Health Commission, and the State Administration of Traditional Chinese Medicine [12]. The National List includes 121 diseases; prioritizing diseases that are characterized with higher prevalence and burden, and that are highly treatable. The creation of these lists has improved awareness and has the potential to lead to further improvements of the treatment and management of rare diseases in China.

### Focus of this study

In our previous work [4], we examined the specifics of the economic burden and described the direct medical costs related to rare diseases in Shanghai. Herein we take a holistic view and focus on rare disease epidemiology in Shanghai. We present epidemiological characterization of a subset of 33 diseases of the Shanghai rare disease list computed from the patient records across local hospitals. Additionally, we focus on the 6 most-common rare diseases of this subset of 33 diseases for further stratification; we show in-hospital expenses and duration of hospitalization. Finally, a period prevalence is calculated for the rare diseases set. Our study’s goal is to contribute to addressing the gap in rare disease knowledge in China; our work is amongst the few that present epidemiology information and report prevalence information regarding rare diseases in China.

## Materials and methods

### Data source

The data sources for the analysis presented in this work were the Health Information System (HIE) [13] of Shanghai and the Shanghai List of rare diseases. The Health Information Exchange (HIE) system of Shanghai, established by Shanghai Hospital Development Center in 2010, integrates medical records from 38 tertiary hospitals, 6 district hospitals and 40 community health centers in Shanghai. The HIE receives a constant stream of new patient medical data and its data contents are constantly expanded; at the time of our data sourcing the HIE system contained information on over 61 million patients with over 210 million visit records, 16 million prescriptions, 9.9 million case notes, and 230 million laboratory results. The HIE systems serves as a foundational element utilized in hospital management activities such as analytics and business operation. In-hospital quality assurance and patient management activities can also be performed utilizing data sourced from the HIE as well as health-related and biomedical research.

### Data extraction and processing

#### Disease list subset

First, we mapped the Shanghai list diseases to standard ICD10 codes from the International Statistical Classification of Disease and Related Health Problems 10th Shanghai Revision. 22 diseases did not have a corresponding ICD10 code and no information could be identified in the HIE, therefore we were able to source patient information for 34 diseases from the Shanghai List. One disease: hypophosphatemic rickets (ICD CODE: E83.308) had no patient records, thus the set of diseases we considered contained 33 diseases.

#### Rare disease patient data

We obtained HIE rare disease medical record data consisting of patient demographic information, patient records (in- and outpatients), prescription information, medical practitioner advice, diagnosis, biomedical indicators, radiology records, and discharge records. Each patient had a major diagnosis and several secondary diagnoses entered in the HIE. Patients with either major or secondary diagnosis were included in our target population. For hemophilia we combined all subtypes: hemophilia (ICD = D66.x02), hemophilia A (ICD = D66.x01), hemophilia B (ICD = D67.x01), hemophilia C (ICD = D68.101).

From the medical records, we sourced a dataset containing the following data-points: patient medical record number, gender, date of birth, consultation date, diagnosis—disease name and ICD10 code, and organization code. For the inpatients we had also hospital admission date, hospital discharge date, and total hospitalization expenses.

To ensure that the privacy and confidentiality of the patients, personally-identifying information was removed before receiving the data. The data was entered into a mySQL database and queried with the database management programs DbVisualizer (DbVis Software AB, Stockholm, Sweden) and by the SQL database query language.

#### Epidemiological Analysis

##### Number of patients, age, gender, outpatient and inpatient proportions, and year-to-year change

Total number of patients per disease per year, age, gender distribution, proportions of inpatients and outpatients were calculated for the 33 diseases for the period January 2013 to December 2016. The total number of patients per disease is a summation of inpatients and outpatients, with duplicate records removed. Patients who have more than one medical record were counted only once, on the basis of the first time of presentation for the year. Age and gender were calculated by summation from the dataset. Age was considered as of the date of first hospital presentation for the year. Inpatient and outpatient proportions were also calculated by grouping from the dataset. The year-to-year change in patient numbers per disease was calculated for each on the 4 years (January 2013 – December 2016).

##### Length of stay and hospitalization expenses

We calculated the average length of hospital stay per disease, in hospital expenses, utilizing inpatient records. Of the 33 target rare diseases, 24 diseases had patients with in-hospital records. We averaged the length of stay in hospitalization by the patient numbers for each disease and the time period of four years. The hospitalization expenses were summed and then averaged by number of patients for each disease.

##### Period prevalence

The period prevalence of the rare diseases was calculated utilizing the following equation:

$${\text{Prevalence}} = \frac{number\,of\,patients\,with\,Shanghai\,hukou\,in\,the\,dataset}{{average\,number\,of\,population\,with\,Shanghai\,hukou\,\left( {2013 - 2016} \right)}}$$

The denominator was set to 14,409,750 people which is the average number of individuals which were registered residents of Shanghai i.e. individuals who possess a Shanghai residence permit (hukou, 户口) for the years 2013–2016. Data was sourced from the Shanghai Municipal Statistics Bureau retrieved from: http://tjj.sh.gov.cn/tjnj/nj17.htm?d1=2017tjnj/C0201.htm.

## Results and discussion

In this work we utilized clinical data from the Shanghai HIE System to characterize of the status of rare diseases in Shanghai. First, we describe the total number of patients, year-to-year change in new patients and the distribution of gender and age for the top six (by patient number) diseases. Second, we described the hospitalization burden in terms of in-hospital ratio, length of stay and medical expenses during hospitalization. Finally, rare disease period prevalence is calculated. The motivation for our work is to present a China focused analysis and a period snapshot of the status of rare diseases in Shanghai. Our work utilizes a dataset that is Chinese sourced, an approach motivated by the potential differences that exist between China and other countries. Our work helps address a knowledge need and gives insights that are pertinent within the domain of China’s health system.

### Total number of rare disease patients for the period 2013–2016 in Shanghai hospitals

**Fig. 1:**
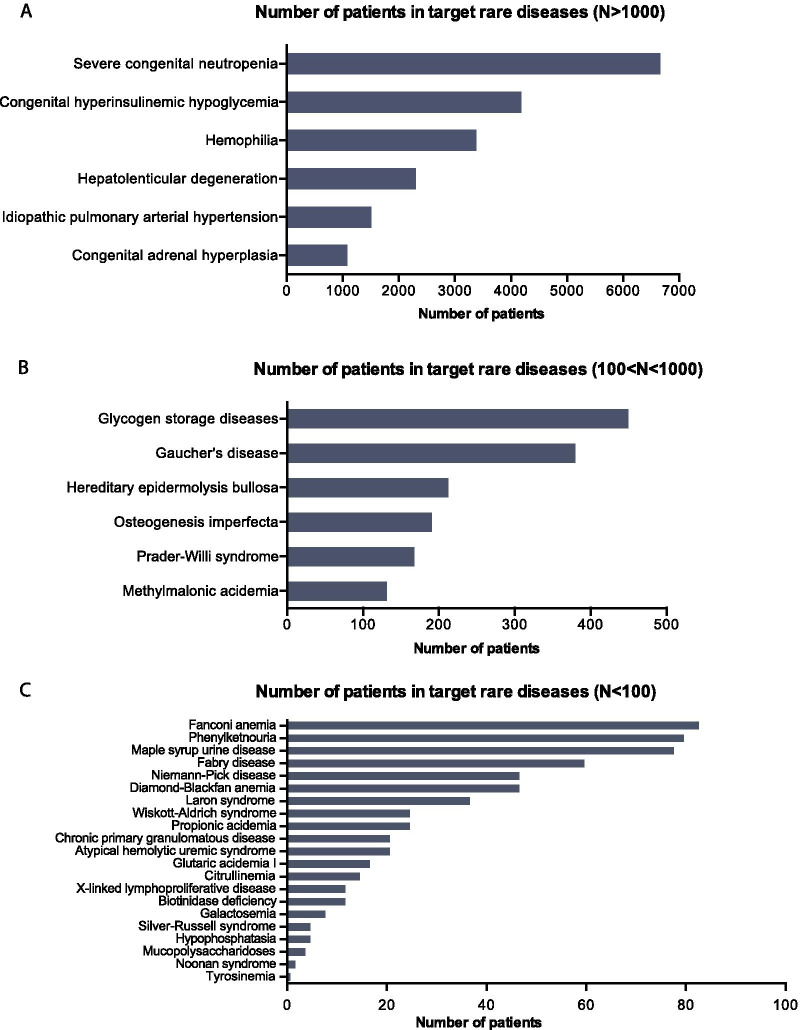
Total numbers of patients diagnosed with one of the 33 rare diseases in Shanghai hospitals for the time period of 2013–2016. **a** Total number of patients for the target rare diseases where N > 1000. **b** Total number of patients for the target rare diseases where 100 < N < 100. **c** Total number of patients for the target rare diseases where N < 100. N indicates the patient numbers

For the period 2013–2016, we identified a total of 16,933 patients diagnosed with a disease from the Shanghai List subset of 33 rare diseases in Shanghai hospitals. Within the subset of 33 diseases, severe Congenital Neutropenia was the most prevalent disease. Patients numbers for six diseases exceeded one thousand (Fig. 1a), six other diseases had patient numbers in the range of 100 to 1000 (Fig. 1b).The remaining 21 diseases had number of patients fewer than 100, and 10 diseases were shown as especially rare in Shanghai with fewer than 20 patients counted during the investigation period of 2013–2016 (Fig. 1c).

### Year-to-year patient numbers change

**Fig. 2:**
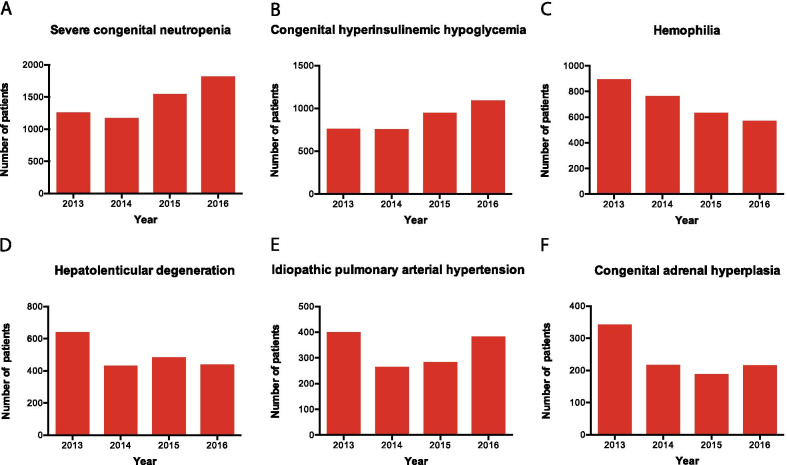
Year-to-year patient numbers change. Year-to-year change in new patients is shown for the six top (by patient numbers) diseases from the Shanghai list. Each red bar depicts the number of new patients each year, (i.e. the number of patients who had a diagnosis for the target disease recorded for them for the first time in that year)

To gain a better understanding of the patient variation year by year, we calculated the number of new patients (patients who had a diagnosis recorded for them for the target disease for the first time that year) per year for the six top (by total patient number) diseases. Severe congenital neutropenia and congenital hyperinsulinemic hypoglycemia showed an apparent increasing trend year by year (Fig. 2a, b), hemophilia showed a decreasing trend (Fig. 2c), and hepatolenticular degeneration, idiopathic pulmonary arterial hypertension, and congenital adrenal hyperplasia showed a variable trend for this four-year period (Fig. 2d–f).

### Age and gender

**Fig. 3:**
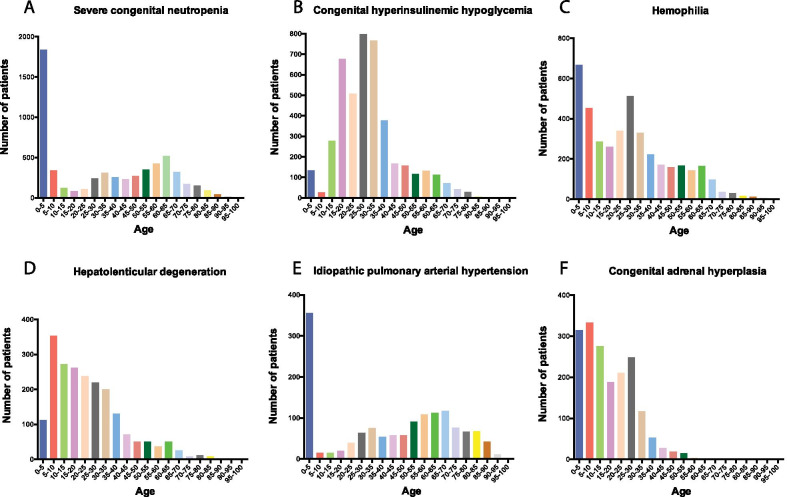
Age distribution of the top six (by patient numbers) diseases. Age axis intervals are equal to 5 years. The age value is taken at the time when the patient had a diagnosis for one of the rare disesases recorded for them for the first time in the HIE system

The distribution of patients by age (Fig. 3) indicates that the majority of the 33 rare diseases of the Shanghai list have patients of young age. For example, in the top six diseases, severe congenital neutropenia and idiopathic pulmonary arterial hypertension showed 33.1% and 26.7% of patients were under 5, respectively. Considering the typical age of onset of the top 6 rare diseases we observed that diseases with early onset such as severe congenital neutropenia show age distribution of diagnosis also skewed towards the early age. One notable difference is seen in Idiopathic pulmonary arterial hypertension which has onset during all ages, whereas it appears to be diagnosed predominantly at early age in the hospitals in the Shanghai HIE network.

**Fig. 4:**
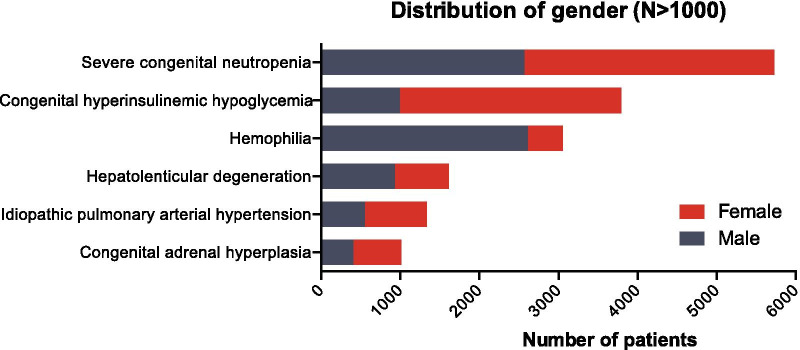
Gender distribution of the top six (by patient number) diseases

The four of top six (by patient numbers) diseases from the Shanghai list have similar numbers of both male and female patients (Fig. 4) which illustrates that there is no evidence of gender bias in rare disease diagnosis in Shanghai hospitals. Amongst the top six rare diseases, two show an imbalanced gender ratio. The male to female ratio of Hemophilia (an X-linked recessive disorder) is 5.92:1 and the male to female ratio of congenital hyperinsulinemic hypoglycemia is 1:2.82. This finding provides further support to the notion of the potential role of X-linkage in the etiology of congenital hyperinsulinemic hypoglycemia [14, 15].

### In-hospital analysis: hospitalization proportion

**Fig. 5:**
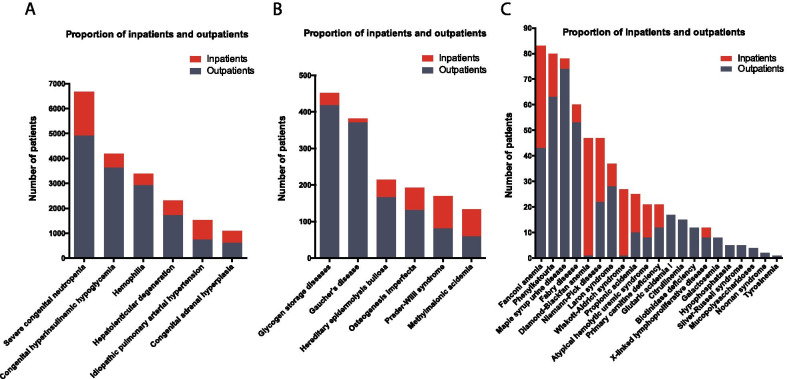
Proportion of inpatients and outpatients in the 33 rare disease subset of the Shanghai List. **a** shows diseases with total number of patients in the thousands, **b** shows diseases with total number of patients in the hundreds, and **c** shows diseases with fewer than 100 patients

There is a notable difference in the numbers of inpatients and outpatients across the 33 rare diseases. Generally, outpatients accounted for a greater proportion in a majority of diseases. In two rare diseases, Diamond-Blackfan anemia and Wiscott–Aldrich syndrome, the number of inpatients greatly outnumbers the number of outpatients. In a few other cases, the proportions of inpatients and outpatients were nearly equal (Fig. 5).

### In-hospital analysis: average length of stay and hospitalization expenses of hospitalization

**Fig. 6:**
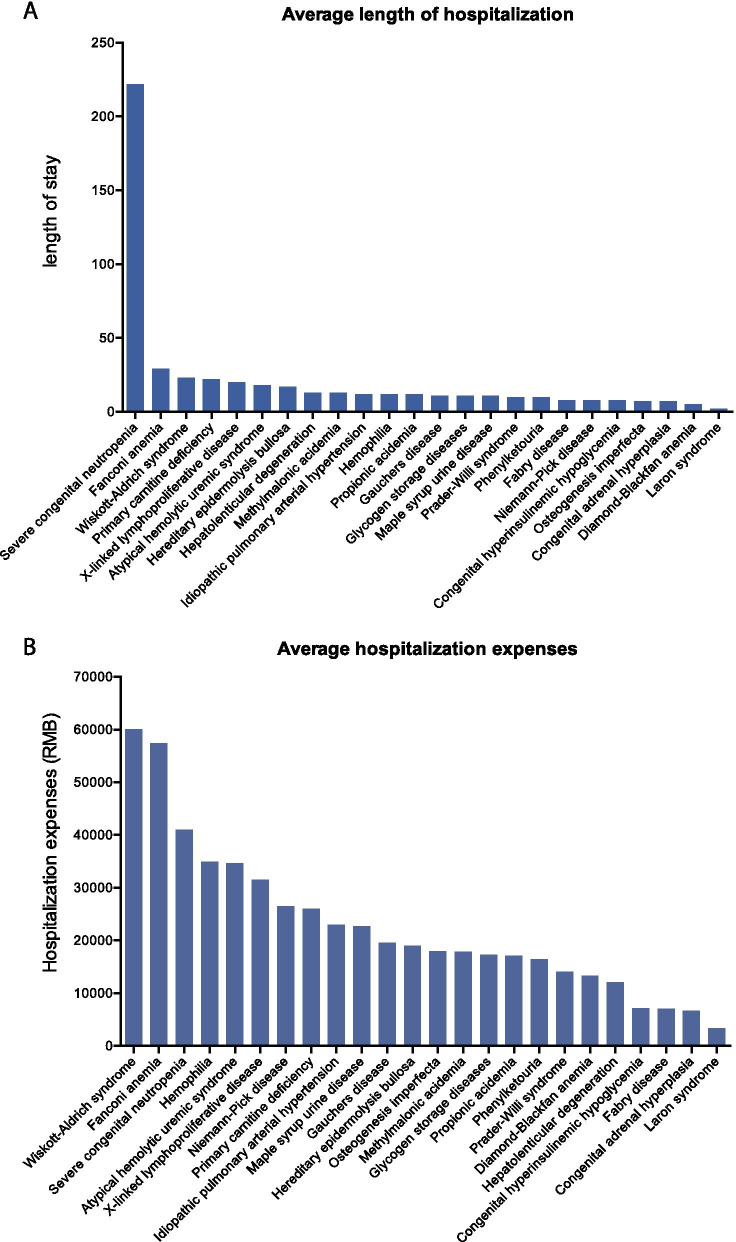
In-hospital analysis: average length of hospitalization and expenses for a 24 rare diseases subset of the Shanghai List. **a** Average length of hospitalization-value shown is the length of stay per person per year for each disease (in days). **b** Average hospitalization expenses per person for each disease (in Chinese yuan CNY)

The majority of the 33 rare diseases had an average length of stay in hospitalization of fewer than 30 days per person every year (calculated over all 4 years) (Fig. 6a). However, the average length of stay for patients diagnoses with Severe Congenital Neutropenia (a disease that is characterized with periods of potentially life-threatening infections [16] that require hospitalization) was 222 days per person in one year (averaged over the 4-year time period). 75.3% of the 33 rare diseases had an average hospitalization stays of more than 10 days. Patients diagnosed with rare disease incurred significant economic burden, and hospitalization expenses ranging from under 5000 CNY to 60,000 CNY – (averaged over the 4-year time period) (Fig. 6b). The top 2 most costly diseases were Wiscoff–Aldrich syndrome and Fancomy anemia with expenses exceeding 50,000 CNY.

### Prevalence calculation

We calculated the period prevalence of rare diseases in Shanghai (Table 1). The 3 most prevalent diseases from the subset of 33 diseases of the Shanghai list were severe congenital neutropenia, congenital hyperinsulinemic hypoglycemia, and hemophilia. The least prevalent disease was primary carnitine deficiency. The prevalence calculation population is the set of people with Shanghai household registration (hukou, 户口), and the set of diagnosed patients with Shanghai hukou (户口). (n = 14,409,750, averaged over the 4-year time period).

**Table 1:** Period prevalence (2013–2016) of 33 rare diseases in Shanghai residents

Rank	Disease name	ICD code	ORPHA code	In National list	Number of patients (Shanghai hukou, 户口)	Prevalence	Period prevalence 1 in 1,000,000 (10^–6^)	Period prevalence 1 in 100,000 (10^–5^)
1	Severe congenital neutropenia [19]	D70.x00	42,738	Yes	3703	2.57E−04	257	25.7
2	Congenital hyper-insulinemic hypoglycemia [20]	E16.103	657	Yes	3337	2.32E−04	232	23.2
3	Hemophilia [21]	D66.x01 D66.x02 D67.x01	448	Yes	931	6.46E−05	64.6	6.46
4	Idiopathic pulmonary arterial hypertension [22]	I27.000	422	Yes	900	6.25E−05	62.5	6.25
5	Hepatolenticular degeneration [23]	E83.001	905	Yes	531	3.69E−05	36.9	3.69
6	Gaucher's disease [24]	E75.201	355	Yes	323	2.24E−05	22.4	0.224
7	Glycogen storage diseases [25]	E74.000	79,201	Yes	218	1.51E−05	15.1	1.51
8	Congenital adrenal hyperplasia [26]	E25.004	418	Yes	196	1.36E−05	13.6	1.36
9	Hereditary epidermolysis bullosa [27]	Q81.900	79,361	Yes	85	5.90E−06	5.9	0.59
10	Maple syrup urine disease [28]	E71.001	511	Yes	57	3.96E−06	3.96	0.396
11	Osteogenesis imperfecta [29]	Q78.000	666	Yes	54	3.75E−06	3.75	0.375
12	Prader–Willi syndrome [30]	Q87.106	739	Yes	33	2.29E−06	2.29	0.229
13	Diamond–Blackfan anemia [31]	D61.001	124	Yes	28	1.94E−06	1.94	0.194
14	Fabry disease [32]	E75.205	324	Yes	17	1.18E−06	1.18	0.118
15	Niemann–Pick disease [33, 34]	E75.203	77,292(A); 77,293(B);646(C)	Yes	17	1.18E−06	1.18	0.118
16	Fanconi anemia [35]	E72.002	84	Yes	16	1.11E−06	1.11	0.111
17	Phenylketonuria [36]	E70.000	716	Yes	14	9.72E−07	0.972	0.0972
18	Methylmalonic acidemia [37]	E71.102	293,355	Yes	8	5.55E−07	0.555	0.0555
19	Laron syndrome [38]	E34.304	633	Yes	7	4.86E−07	0.486	4.86
20	X-linked lymphoproliferative disease [39]	D82.301	2442	Yes	5	3.47E−07	0.347	0.0347
21	Atypical hemolytic uremic syndrome [40]	D59.301	2134	Yes	4	2.78E−07	0.278	0.0278
22	Propionic academia [41]	E71.101	35	Yes	4	2.78E−07	0.278	0.0278
23	Wiskott–Aldrich syndrome [42]	D82.000	906	Yes	4	2.78E−07	0.278	0.0278
24	Hypophosphatasia [43]	E83.306	436	Yes	3	2.08E−07	0.208	0.0208
25	Biotinidase deficiency [44]	D81.801	79,241	Yes	1	6.94E−08	0.0694	0.00694
26	Primary carnitine deficiency [45]	E71.302	158	No	1	6.94E−08	0.0694	0.00694
27	Citrullinemia [46]	E72.202	187	Yes	0	n/a	n/a	n/a
28	Galactosemia [47]	E74.201	352	Yes	0	n/a	n/a	n/a
29	Glutaric acidemia I [48]	E72.302	25	Yes	0	n/a	n/a	n/a
30	Hypophosphatemic rickets [49]	E83.308	437	Yes	0	n/a	n/a	n/a
31	Mucopolysaccharidoses [50]	E76.000	79,213	Yes	0	n/a	n/a	n/a
32	Noonan syndrome[51]	Q87.105	648	Yes	0	n/a	n/a	n/a
33	Silver–Russell syndrome [52]	Q87.100	813	Yes	0	n/a	n/a	n/a
34	Tyrosinemia [53]	P74.501	3402	Yes	0	n/a	n/a	n/a

Evidence-based prevalence metrics calculations inform health policy and also serve as establishing or updating existing rare-disease definition by establishing a population threshold [17]. The lack of available rare disease epidemiology data in China has fostered the development of other proposed approaches—such as the Shanghai List or other orphan drug economics-focused methods [18] and in general such prevalence calculations in the Chinese context have been scarce. The prevalence results presented in this work are one of the few available epidemiology data based calculations focused on one of China’s first-tier cities and can serve as a landmark evidence in rare disease research and policy development.

## Summary and conclusion

Increasing knowledge and awareness of rare diseases for health care providers and the general public, ongoing work on developing new treatments and ensuring drug access, and fostering governmental policy implementation are some of the key aspects that will help drive the successful management of rare diseases.

In this work the status of rare diseases in Shanghai, China is characterized. Total number of patients, year-to-year change in new patients and the distribution of gender and age for the top six (by patient number) diseases, together with the hospitalization burden in terms of in-hospital ratio, length of stay, and medical expenses during hospitalization are described. A rare disease period prevalence calculation for 33 rare diseases in Shanghai is presented. The work covers the period 2013–2016 and it is the authors’ goal to provide a 7-year update publication at a future date.

## Data Availability

Not applicable.
